# Signatures of movement variability anticipate hand speed according to levels of intent

**DOI:** 10.1186/1744-9081-9-10

**Published:** 2013-03-06

**Authors:** Elizabeth B Torres

**Affiliations:** 1 Psychology Department, Rutgers Center for Cognitive Science, Rutgers Computational Biomedicine Imaging and Modeling Center, Rutgers University, 152 Frelinghuysen Rd, Piscataway, NJ, 08854, USA

**Keywords:** Intended movements, Incidental movements, Speed variability, Stochastic rule

## Abstract

**Background:**

Complex movement sequences are composed of segments with different levels of functionality: intended segments towards a goal and segments that spontaneously occur largely beneath our awareness. It is not known if these spontaneously-occurring segments could be informative of the learning progression in naïve subjects trying to skillfully master a new sport routine.

**Methods:**

To address this question we asked if the hand speed variability could be modeled as a stochastic process where each trial speed depended on the speed of the previous trial. We specifically asked if the hand speed maximum from a previous trial could accurately predict the maximum speed of a sub-sequent trial in both intended and spontaneous movement segments. We further asked whether experts and novices manifested similar models, despite different kinematic dynamics and assessed the predictive power of the spontaneous fluctuations in the incidental motions.

**Results:**

We found a simple power rule to parameterize speed variability for expert and novices with accurate predictive value despite randomly instructed speed levels and training contexts. This rule on average tended to yield similar exponent across speed levels for intended motion segments. Yet for the spontaneous segments the speed fluctuations had exponents that changed as a function of speed level and training context. Two conditions highlighted the expert performance: broad bandwidth of velocity-dependent parameter values and low noise-to-signal ratios that unambiguously distinguished between training regimes. Neither of these was yet manifested in the novices.

**Conclusions:**

We suggest that the statistics of intended motions may be a predictor of overall expertise level, whereas those of spontaneously occurring incidental motions may serve to track learning progression in different training contexts. These spontaneous fluctuations may help the central systems to kinesthetically discriminate the peripheral re-afferent patterns of movement variability associated with changes in movement speed and training context. We further propose that during learning the acquisition of both broad bandwidth of speeds and low noise-to-signal ratios may be critical to build a verifiable kinesthetic (movement) percept and reach the type of automaticity that an expert acquires.

## Introduction

Motor variability has emerged as an important component of movement control research, informative of learning and optimization strategies in the nervous system [1-9]. The relevant roles of movement variability in the development of motor strategies was pointed out by Bernstein who observed that we do not perform the same movement exactly the same way twice [10]. Such inherent variability in our motions contributes during early development to the formation of a motor percept [11] that assists us in transitioning from spontaneous movements to goal-intended actions under voluntary control [12-16].

**Figure 1:**
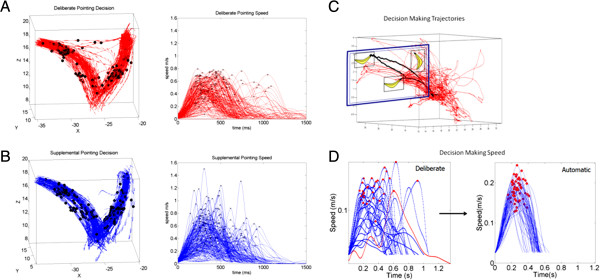
**The stability of the unimodal instantaneous speed profile in point to point hand movements and its susceptibility to changes in task context, decision making, and cognitive loads make the velocity dependent parameters a good candidate to amplify motor variability in deliberate vs. spontaneous control.** (**A**) Velocity flows towards two alternative choices during a decision making task initiated from a similar position. Horizontal flows are from “change of mind” trajectories midway to the target. Black dots mark the place along the trajectories where the maximum velocity occurred. Speed profiles from the velocity flows are very variable and never in this task get unimodal. (**B**) the retractions of this task are also variable as the decision making process continues to unfold even after the decision was made (data from one graduate student out of 6 with similar performance presented at [23,24]). (**C**) The hand trajectories of a child performing a pointing gesture to communicate his decision of a match to sample task. This is what natural pointing movements look like. (**D**) The evolution of the hand trajectories and decision making of that child captured and amplified in the speed profiles and the speed maxima (red profile is the forward segment of the black trajectory towards the target). Notice the regained stability of the hand speed which turns unimodal again within minutes.

Upon maturation of voluntary reaches we seem to develop a stable speed profile in point to point hand movements [12-16] characterized by a single peak. This signature is maintained in adulthood [17,18]. Yet the unimodal profile is also impacted by the task’s context and can be flexibly reshaped on demand [19,20]. See for example (Figure 1) where pointing motions in the context of decision making are impacted in a young adult (A) and in a 5 year old child (B). The stable unimodal profile generally recovers from such alterations [19,20] unless the system is compromised by stroke or neurodegenerative disorders [21,22]. In this regard the variability of velocity-dependent parameters such as the maximum speed, the maximum acceleration and their timing may serve as an amplifier of somatosensory processes.

The empirical frequency distributions of velocity-dependent parameters *as the movement unfolds* are not generally known. The bulk of motor control research in point to point behavior has rather focused on the spread of spatial errors at the endpoint of the reach, after the motion trajectory has been completed and the hand has landed on the target [5,7,25-27]. Even when the spatial error spread may be stable, the temporal variations of that spread may be large (Figure 1A). Likewise a very automatic tempo may accompany a large spatial spread when motions are naturally unconstrained (Figure 1B). Very little is known about the statistical properties of the spatio-temporal aspects of the kinematics parameters during the performance of the action, as the movements unfold. Unveiling the statistics of velocity dependent parameters of the hand in flight may be useful to gain a better understanding of our kinesthetic abilities to adapt old motor programs to new contexts. In sports adaptation is commonplace as athletes often experiment with different training regimes to gain effectiveness and optimal timing of their movements.

Perhaps in complex sports routines that include point-to-point segments, the stability of the speed maxima and its sensitivity to movement context could help us assess repetitive training performance as a stochastic process over time. In this context we propose that the fluctuations of such velocity-dependent trajectory parameters can be understood as *reentrant* sensory information in the system: a form of kinesthetic input that at the motor output gives a readout of our somatosensation, its reliability, its flexibility or its persistence (as when the system shows persevering after-effects induced by force perturbations [28] or by changes in the geometry of motion trajectories [19]).

The fluctuations inherently present in the ongoing movements of our limbs as they unfold in concert with other body parts could serve as an important source of information to model human behavior as a stochastic process. From trial to trial such minute fluctuations in the motion trajectories of our body and limbs may also play an important role in helping us anticipate impending performance, acquire better priors and flexibly reshape our existing motor programs according to new contexts and new demands. From repetition to repetition of the same task these minute fluctuations could be thought of as re-afferent micro-movements. In this sense as much as the spread of the spatial error at the target may contribute to the use of priors in the context of Bayesian inference [4,7,25,29,30]; or to the minimization of certain costs in the context of optimization theories [4-6,29]; or to the overall improvement of centrally-driven learning-adaptation strategies [18], so might the kinesthetic input that comes from the periphery and that is sensed as the movement unfolds. This input forms a spatio-temporal kinesthetic percept that must be integrated with other forms of sensory feedback. Yet how the statistics of this percept may change in real time, during training, is unknown, particularly in complex sports actions, across different training contexts. In such actions intended and supplemental motion segments coexist and have distinct stochastic somatosensory signatures [31,32].

Besides varying their speed, during training, athletes in contact sports (such as boxing and karate) use other means to impact repetitive behavior and get faster, more effective and more accurate at delivering their strikes. These variations include loads, mirror-feedback and even training in complete darkness. It is unknown what the role of supportive motions that are incidental to the main goals may be under such different contexts. In particular it is unknown what role the velocity-dependent variability may play under such different training contexts.

Motivated by our recent results and by previous work indicative of the role of motor variability in anticipatory strategies of intended behaviors here we explore the statistics of velocity dependent parameters in supportive motions of martial arts routines across experts and novices. From the empirical data we estimate the statistical properties of velocity dependent parameters as the motions unfold and examine anticipatory and learning performance in novices and experts under different training contexts.

## Methods

The experimental methods and apparatus used in this paper are described elsewhere [31]. Participants included a second-degree black-belt martial arts expert (23 years old), a first-degree black-belt martial arts expert (22 years old) and 13 novices (ages 19–29 years old.) All 15 participants (6 females and 9 males) were members of Rutgers the State University of New Jersey, USA. The Rutgers University Institutional Review Board in compliance with the Declaration of Helsinki approved the protocol for the movement studies. Consent for videotaping was obtained from the participants.

**Figure 2:**
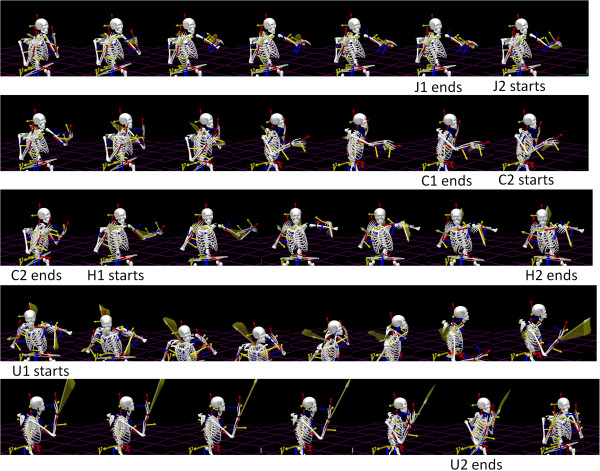
**Full routine breakdown according to upper limbs’ motions.** Rendering of a subject’s upper body and extremities with axes measuring changes in position and orientation of the limbs, head and trunk. The Jab forward (J1) ends as the retraction (J2) starts simultaneously with the Cross forward (C1). This is followed by the retraction of the Cross (C2) which rotates the body and simultaneously initiates the Hook forward (H1). The helical axes in light yellow (spanning a fan of vectors) show relative rotations between two coupled body parts. The length of that vector is proportional to the net coupled rotation. They are really evident during the Upper Cut. The focus of the paper is on the Jab both performed in isolation and embedded in the full routine shown here.

We used 16 electromagnetic sensors (240 Hz Polhemus Liberty) to measure the motions continuously. Fifteen sensors were attached to the participants’ body and one sensor was used to digitize the body and build a biomechanical model using commercial software (The Motion Monitor, Sports Inn). The software filtered and smoothed the position data and provided first order rate of change of displacements (linear velocity) to yield the parameter of interest in this study, the maximum speed of the trajectories (m/s). It also provided second order rate of change, linear acceleration (m/s^2^) to assess possible rules connecting the stochastic trajectories of these two interdependent parameters over time in one session. The full routine is shown in Figure 2.

This paper focuses on the motions of the hand during the forward and retracting Jab, which was extracted from the speed profiles of the hand trajectories [31] under two instructed speeds (slow and fast) and for different training contexts. We report the variability patterns in the Jab (performed in isolation and embedded in a complex sequence, 100–120 trials minimum per subject-speed condition).

To determine the speed level for each individual participant we instructed the participant before the experiment to move at his/her comfort speed (normal) and to perform faster or slower motions relative to that level. We used a chronometer to measure for each subject the duration of the single routine and of the combinations under his/her self-determined speed level. This time window was then used to assist us in the data collection so as to allocate enough recording time to buffer each trial. During the experiments participants received no feedback on their speed performance.

Participants consisted of 2 groups. The first group (previously described in [31]) performed sequences of martial arts routines (Jab-Cross-Hook-Uppercut) at different speeds. They also performed the sub-routines in isolation (i.e. the full forward and back Jab loop, etc). The speeds were called at random using computer generated sequences of fast and slow levels. Members of this first group (6) also performed a minimum of 10 trials under each one of 6 different contexts listed below to probe the effects of sensory guidance on movement variability. The contexts included:

1. *Simulation*: Subjects were instructed to perform the routine from memory without guidance from the instructor, as if an opponent was present.

2. *Mirror feedback*: Subjects were instructed to perform the routine in front of a mirror and use the feedback from the reflection of their body on the mirror.

3. *Dark with eyes closed*: The lights in the room were turned off and subjects were instructed to close their eyes and perform the routine in complete darkness. To ensure compliance a bandana was used to cover the eyes.

4. *Loads*: Subjects carried training loads on both of their arms (12 lbs in each forearm). The loads were distributed along the forearm and consisted of 3 lbs sand bags with Velcro that secured the loads to the forearms. These small sand bags are commonly sold at sports stores for training.

5. *Mirror with body lights*: Subjects were instructed to perform the routine in the dark, in front of a mirror with glowing sticks attached to the body (using Velcro). They were instructed to use visual feedback from the reflexion of the body lights on the mirror. These resembled a simplified version of the body (as a stick figure with point-lights).

6. *Body lights*: Subjects were instructed to perform the routine in the dark with glowing sticks placed on the body but no mirror. Subjects were instructed to use feedback from their glowing sticks as much as they could.

The second group (9 participants) performed combinations of Jab-Cross-Hook-Upper Cut in a block-design of fast or slow speeds. Each block had one speed only. As in the first group, the full Jab (strike and retraction) was performed embedded in the full routine and as a sub-routine in isolation. This group had two experts to whom we compared novices against. In this group, besides simulating strikes against an imaginary opponent, we also studied in 4 participants (2 experts and 2 novices) the actual punches against a training bag. The bag was held by another participant. This control experiment enabled us to further test the velocity-dependent variability of the unfolding hand motions in different training conditions: training to actually hit the target at different speeds against training that did not hit the physical target. We asked to what extent the probability distributions of velocity-dependent parameters that we empirically estimated shifted for novices vs. experts across training contexts.

The bulk of the paper will focus on the Jab subroutine and the simulation condition across the 15 participants including the 2 experts in martial arts. We will also assess the statistics of the other 5 conditions in a smaller subset of 6 who participated in such experiments; and those of the physical bag vs. imaginary target in a small sub-sample of 4 participants (2 experts and 2 novices).

## Motivation for the methods used in the present study

It is important to notice that this was an exploratory study with no particular expectations, other than finding out what the actual empirically derived probability distributions of various velocity-dependent parameters were for this routine. The study was also aimed at understanding if there were systematic stochastic differences between the intended and spontaneous sub-movements of the Jab. The Jab is a motion particular to martial arts and contact sports (karate, boxing, etc). However its basic forward and retracting structure is also present in the commonly studied reaching or pointing behaviors. Motor control experiments focus only on the forward segments towards the target. In recent years we took an interest in the retractions as well [19,22] because they can be more revealing of a break down in the balance between automatic and voluntary control than the forward segments intended to the target tend to be. Spontaneous retractions amplify the movement trajectory variability in such a structured way that one can blindly extract contextual information and movement type from them [31].

The main drives of the work were (1) to assess if velocity-dependent stochastic metrics were different for different levels of speed and different training contexts; and (2) if these differences between movement functionality (i.e. goal-directed vs. supportive) were manifested differently in novices and experts; or if despite the disparity in practice level, some commonality could be found. The first question more precisely asks if there is a conservation of some velocity-dependencies from trial to trial despite instructed speed levels and differences in training contexts that may systematically change when the motion is incidental. The second question addresses if common strategies may be inherently present in the spontaneous retractions of both the expert and novice systems, independent of training levels.

## Analytical methods

We use a type of distributional analysis that we recently developed [31,32] in a collaborative effort [33] to assess the empirical frequency distributions of velocity-dependent parameters in the trajectories of natural movements [34]. Using these techniques we have developed biomarkers to automatically classify severity in spectral disorders such as Autism and Parkinson’s disease [35] and to treat somatosensory-motor aspects of these disorders [36,37].

Here we obtained the empirical frequency distribution of each parameter of interest for each subject and used the continuous Gamma probability distribution family to assess the best parameter estimates for each subject. In previous work we had found that this family captures the whole range of human behavior well, along the spectrum of human somatosensation, including autism, Parkinson’s disease and patients with deafferentation or stroke in the left Posterior Parietal lobe [21,32,38]. Thus while the kinematics data of interest in healthy adults is well described by the log-normal distribution [31] the log-normal fails in immature or compromised systems [21,32], and one must look into other families. The continuous family of probability distributions obtained through different values of the parameters of the Gamma probability distribution captured with high confidence all the somatosensory-motor ranges across ages and clinical populations.

It is important to note that, unlike traditional significance hypothesis testing methods, our new methodology does not assume homogeneity of the sample under a common probability distribution. Rather this methodology allows us to study the individual. It also addresses the heterogeneous nature of the kinesthetic/somatosensory variations across a population. Thus, instead of grouping subjects a-priori to assess treatment outcomes or effects within and between groups, the present method uncovers the probability distribution for each individual subject that best characterizes his or her inherent micro-movements’ variability as reflected in velocity-dependent parameters (for example). Then, any commonality in the sample will automatically aggregate to form self-emerging clusters within a large cohort. Such automatically formed clusters are thus indicative of different statistical classes according to the somatosensory read out from the person’s micro-movements.

In the context of sports learning this clustering can be informative of adequate individualized training regimes to accelerate learning or maximize effectiveness, etc. Perhaps what works well for one athlete will not work as well for another athlete. Somatosensory signatures can tell us the progression in novices and in experts. The use of experts is thus a point of reference to assess what types of scenarios one may expect to have in the limit. The experts in this study, for instance, had practiced these routines over 10,000 trials minimum across years of training and teaching others. Yet the velocity-dependent parameters revealed subtle changes with training context according to our new metrics. Variability in the novices can also be very precisely quantified via changes in the noise to signal ratios, both when motions are deliberate and when they carry spontaneous fluctuations.

In brief, because of the underlying assumptions of significance hypothesis testing, which homogenize the behavioral data a priori and test the null hypothesis, those traditional methods cannot capture systematic subtle differences in somatosensation as these fall under different probability distributions [32]. Trying to homogenize the sample can be problematic. This is particularly so if one’s goal is precisely to learn about those subtle differences that the traditional approaches tend to wash away.

**Figure 3:**
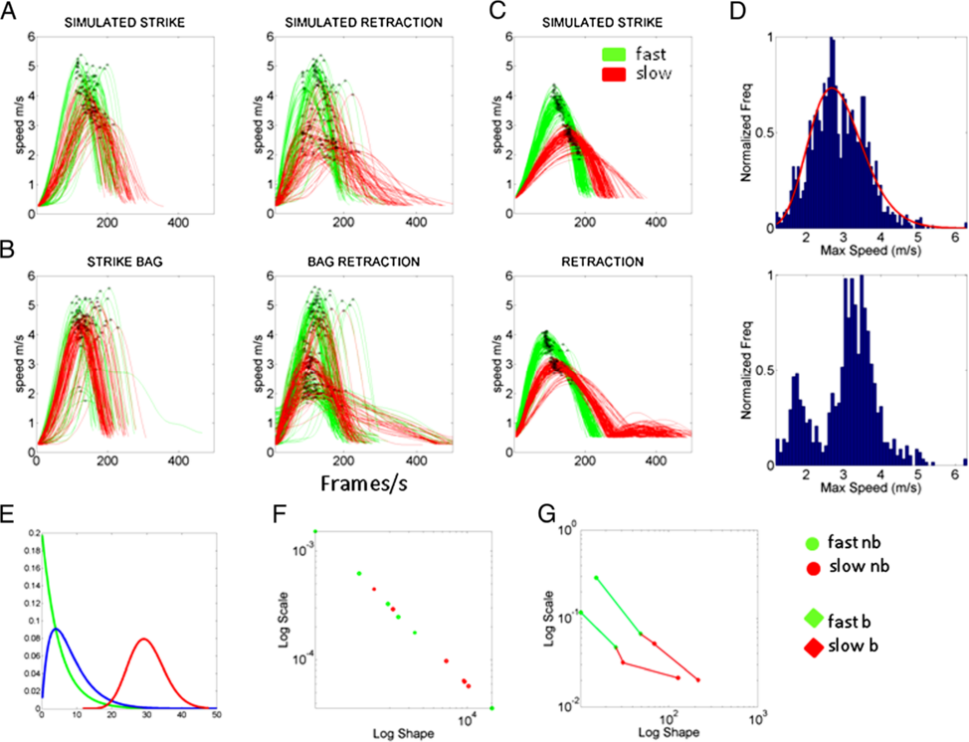
**Analytical methods.** (**A**) Representative hand’s instantaneous speed profiles during the Jab-strike (left) and retracting-Jab (right) in the block of simulated opponent under randomly instructed speeds. Sampling resolution of 240Hz, movements lasting between 0.8s and 2.1s) (**B**) Same as A but striking against the physical punching bag and retracting from it. (**C**) Same as A, but with instructed speeds in a block design. (**D**) Empirical frequency distributions of the ensemble data from A (randomly instructed speeds on top) and speeds from the block design (bottom). (**E**) The continuous Gamma family probability density function curves across a subset of values for the shape and scale parameters in the legend. (**F**) The plots of some subjects for fast and slow speeds (simulated and punching-bag intermixed) using the normalized maximum velocity and estimating the stochastic signatures of each condition. The log-log plot of the shape and scale plane aligns the points along the line of unity. (**G**) The stochastic signatures dynamically measured in real time: stochastic trajectories of intended movements for two subjects across different training contexts with 110 trials each (fast-bag, fast-no-bag, slow-bag, slow-no-bag) measuring predictability towards the right extreme (Gaussian range of the Gamma plane) and randomness towards the left extreme (Exponential range of the Gamma plane).

The histograms and estimation of bin size for the parameters of interest were obtained using MATLAB routines developed in-house based on well-established algorithms for optimal estimation with W=3.49σN−13[39] where *W* is the width of the bin, σ the standard deviation of the distribution (we used estimated standard deviation *s*) and *N* is the number of samples. Figure 3A-Methods shows examples from the same participant of hand speed profiles with the peak velocity highlighted in black. These were gathered across a session where the speed was called at random during the simulated condition of striking an opponent Methodsand spontaneously retracting the hand as the cross strike initiated. Figure 3B shows the same subject when the strike was directed towards a physical target (a punching bag held by the opponent).

When the task is blocked so that the instructed speed is the same within one block and each speed block is randomly selected, the distinction is more evident. This is depicted in Figure 3C for one of the experts during simulated Jab. Figure 3D top panel shows sample normalized frequency histogram from the maximum speed from experimental version in A, obtained by merging all randomly called trials across the block. The bottom panel of Figure 3D is the frequency histogram of the maximum speed when taken across all trials in the different blocks of the block design version. This non-unimodality was significant (p < 10^-15^, dip 0.026) according to the Hartigan’s dip test of unimodality [40]. Obtaining this measurement was important as the distributional analyses detailed below require the distribution to be unimodal. Thus, in the second blocked version of the experiment we group trials per instructed speed within each given speed block and ask if there are shifts in the stochastic signatures across blocks. In the randomly called case, we are rather interested in self-emerging clusters of speed level as the somatosensory signatures of the novices begin to distinguish and anticipate the differences in noise patterns corresponding to different randomly called speed levels. The stochastic maps that we later study are thus more interesting in the cases where the speed level is randomly called. The block design is more interesting to address contextual training and fatigue effects within a given speed.

Other velocity dependent parameters can be assessed as well in the angular domain, (e.g. the time to the maxima, the displacement or rotational distances traveled up to the maximum, etc.) but those analyses are beyond the scope of this paper. Importantly, we also normalized the speed maximum per trial by dividing it by the sum of the speed maximum and the averaged trial speed. This normalization removes allometric effects of different body-size across participants. This is a metric commonly used in analyses of anthropological data [41]. Whereas the maximum speed serves to visualize individual effects, the normalized data serves to inform us about group effects. It is worth noting that each kinematic parameter may be more or less informative about the phenomenology under study. It is up to the researcher to choose which one to use based on the task and quest. In our task velocity was one of the manipulated parameters, and one that varies with context as the movement unfolds. Thus we used velocity dependent parameters in the linear displacement domain. For analyses of the Upper Cut and Hook velocity dependent rotational parameters may be more informative -as suggested by the helical axes indicating large relative rotations between body parts in Figure 2.

The probability density function of the Gamma distribution used to fit speed data of both intended and unintended motions for each individual is given by:

(1) $$y=f\left(x|a,b\right)=\frac{1}{b^{a}\Gamma \left(a\right)}x^{a-1}e^{-\frac{x}{b}}$$

with shape (a) and scale (b) parameters and the Γ function. By varying the shape and scale parameters, one can go from the random, “memoryless” Exponential distribution to the predictive, symmetric Gaussian range of the Gamma plane. This is illustrated in schematic form for a narrow range of values for each of the shape and scale parameters in Figure 3E.

The fluctuations across trials from the movement parameters of interest for each individual have distinct stochastic signatures. The empirical frequency distributions of such parameter can be used to estimate the Gamma parameters that uniquely label that individual’s somatosensation as (a,b) on the Gamma-plane using maximum likelihood estimation (MLE). Thus the MLE point represents a stochastic signature for an individual subject under a certain training context. Across subjects the scatter of points thus obtained forms a “proprioceptive map” of that velocity-dependent parameter. Yet this is a static snapshot of the individual’s somatosensation under one task condition or training context. An example in Figure 3F is shown for some subjects under different speeds and training contexts, just as an example of the normalized parameter that aligns according to an exponential relation between the shape and the scale. The log-log plot aligns the scatter along the line of unity. We can see this by using the general model *f*(*x*) = *n* · *x*^*m*^. We used the MATLAB curve fitting toolbox to assess the goodness of fit in the case of the Gamma plane and also in the case of the first order stochastic rule that we studied.

Later we will take a snapshot at all 15 subjects. Figure 3G on the other hand uses the variability across trials of the individual’s maximum velocity shown for 2 subjects. Here we show how to trace in real time the progress of each individual for a training session. The legend depicts the conditions. For each subject we start out with fast-no-bag (simulated opponent), then switch to fast-bag, then to slow-no-bag and finally to slow-bag. These two subjects have fairly similar stochastic trajectories yet that is just a coincidence. The point is that we can dynamically track the stochastic trajectories of the somatosensory signatures of each person as a function of task context, cognitive demands, levels of automaticity, fatigue, etc. This can be done in real time (every 30 minutes for instance) and/or longitudinally across multiple sessions. The lines connecting the four locations in Figure 3G indicate a shift in the stochastic signatures of a velocity-dependent movement trajectory parameter (the maximum linear speed value). Other parameters could also be used to dynamically track the subject’s performance.

The shift from condition to condition is unique to each person. A shift to the right indicates positive gain, a change towards the Gaussian (symmetric) range of the Gamma with higher predictive power than a shift to the left. A shift to the left indicates negative gain—towards the “memoryless” Exponential range. In a process characterized by the Exponential, events have no predictive power (prior movements are not contributing in a systematic and predictive way to future movements). Sample pdf curves towards the Exponential and towards the Gaussian (the two limiting cases) are illustrated in schematic form in Figure 3E for (a,b) as (1,4) in red and (10,4) in magenta respectively.

These real-time stochastic trajectories permit us to sort through the movement’s spontaneous fluctuations and find the stimuli that best shift the person’s somatosensation towards a verifiable percept with predictive power. In the context of sports we can then see which training regime/context is best for a given athlete and personalize the training schedules.

This methodology is general. It works for complex sports routines or for simpler pointing behaviors. More importantly it is possible to funnel out of the spontaneous fluctuations of the motions the best type of stimuli for each person. These could include different sources of sensory guidance, so as to identify which sensory stimuli would result in more predictive behavior. We can thus readily quantify the shifts towards predictive ranges and build stochastic trajectories that would *optimally* lead the person towards the proper regime of predictability. This is like building a personalized cost function empirically, through the empirical approximation of its stochastic somatosensory *gradient*. This method is inspired in our previously derived partial differential equation in [19,42] yet empirically adapted to the stochastic regimes of biological motions.

## Trajectory analyses and fatigue effects

To address possible effects of speed and loads on the curvature of the trajectories we used a simple linearity metric commonly used in analyses of three dimensional trajectories [19]. This metric approximates the actual theoretical computation of curvature [43] which has a jerk term in the denominator. The filters of the Motion Monitor Inc. software handle velocity and acceleration well but we should not trust jerk (the rate of change of acceleration) from any smoothing procedure because instrumentation errors get amplified, so we use this *linearity* approximation.

The trajectories from the hand motions were resampled to have the same number of frames equally spaced (time-normalized curves.) Upon resampling one must make a super-imposed plot of the original trajectories and the resampled ones to ensure that the resampling procedure maintained the original curves. Each point along the resampled trajectory is now uniformly spaced and projected at a right angle onto the Euclidean straight line. The length of the deviation from the line (related to the amount of bending of the curve) is obtained and plotted as a function of the number of frames. The empirical frequency distributions of this trajectory parameter tend to be skewed. Non-parametric statistics are used (Kruskal-Wallis test) to assess significant differences as a function of speed and speed plus loads.

To address whether fatigue was a significant contributing factor (as it always is in long training sessions) we examined the first 10 trials and the last 10 trials of each block where the speed was the same and compared the maximum speed values and the duration of the motion. It is worth noticing that subjects refused to take breaks as the motions were rather simple. The 100 trials went by very fast. Often spatio-temporal parameters are indicative of differences between early and late times within a session which may relate to fatigue. Regardless of statistical significance we anticipate fatigue as a contributing factor to the overall noise-related results in actual training.

## Results and discussion

In this section of the paper we report and discuss the results from the distributional analyses, the stochastic maps and the overall effects of training context and fatigue.

### Effects of speed and training context on the kinematic parameters of movement trajectories

The instructed speed modulated the velocity of the motion across participants. Novices had no problem distinguishing speed level across blocks of instructed speed, yet within the same block when the speed was instructed at random, their motions tended to blur the difference between speed levels. Experts’ motions delineated speed very well regardless of experimental condition (random vs. blocked). Ranges and median of speed maxima are reported on Table 1 along with *p-values* from the Wilcoxon ranksum test on speed maxima for experiment type and training context. Notice the different effects of training context on the levels of maximum speed. Notice also that in all conditions where the subjects were specifically asked to pay attention to their motions as they were reflected in the mirror, or as the glowing sticks reflected light in the dark, the distinction between strike and retractions became blurred. This was particularly evident in the fast motions. The instruction to attend to the full loop of the routine and use the visual feedback to correct the motions changed the spontaneous nature of the retractions. This effect was captured in the statistics of the maximum speed which rendered the strike and retractions indistinguishable.

**Table 1:** Median values and ranges of the maximum speed across participants in each group and training context

**Training context**	**Subject group**	**Median (min-max range)**	***p-value***
			**Wilcoxon test**
Slow-Fast random (simulation)	6 subjects	Strike 3.56 (0.63, 10.04)	**Strike vs. Ret**
		Retract 2.50 (0.50, 8.14)	1.0483e-004
Slow (dark)	6 subjects	Strike 1.53 (0.50, 2.36)	**Fast vs. Slow**
		Retract 1.40 (0.52, 3.24)	3.4990e-008
			**Slow Strike vs. Ret**
			0.3973
Fast (dark)	6 subjects	Strike 2.24 (0.96, 2.88)	**Fast Strike vs. Ret**
		Retract 1.77 (1.26, 2.49)	0.049
Slow (loads)	6 subjects	Strike 2.43 (1.51, 4.19)	**Fast vs. Slow**
		Retract 2.58 (1.01,4.17)	5.6133e-043
			**Slow Strike vs. Ret**
Fast (loads)	6 subjects	Strike 4.04 (2.51, 6.08)	0.9293
		Retract 4.05 (1.59, 6.31)	**Fast Strike vs. Ret**
			0.0131
Slow (glowing sticks body)	6 subjects	Strike 2.07 (1.49, 2.64)	**Fast vs. Slow**
		Retract 2.56 (1.65, 2.97)	2.6748e-024
			**Slow Strike vs. Ret**
Fast (glowing sticks body)	6 subjects	Strike 4.19 (3.20, 5.34)	1.5773e-007
		Retract 3.75 (3.30, 4.53)	**Fast Strike vs. Ret**
			0.019
Slow (mirror)	6 subjects	Strike 1.87 (0.51, 2.25)	**Fast vs. Slow**
		Retract 1.54(0.50, 2.41)	0.883
			**Slow Strike vs. Ret**
Fast (mirror)	6 subjects	Strike 1.53 (0.51, 2.51)	0.09
		Retract 1.61 (0.52, 3.05)	**Fast Strike vs. Ret**
			0.43
Slow (glowing sticks mirror)	6 subjects	Strike 1.83 (100, 2.34)	**Fast vs. Slow**
		Retract 1.61 (1.23, 3.09)	**0.002**
			**Slow Strike vs. Ret**
			0.317
Fast (glowing sticks mirror)	6 subjects	Strike 2.23 (1.19, 3.46)	**Fast Strike vs. Ret**
		Retract 1.71 (1.51, 3.32)	0.127
Fast block	9 subjects	Strike 3.19 (0.64, 8.31)	**Fast vs. Slow**
		Retract 2.90 (0.52,. 5.36)	7.1035e-059
			**Slow Strike vs. Ret**
			4.1993e-017
Slow block	9 subjects	Strike 2.43 (0.72, 6.54)	**Fast Strike vs. Ret**
		Retract 2.03 (0.31, 4.60)	1.1409e-009
Slow bag	4 subjects	Strike 1.08 (0.26, 5.11)	**Fast vs. Slow**
		Retract 3.06 (0.22, 4.94)	9.7665e-038
			**Slow Strike vs. Ret**
Fast bag	4 subjects	Strike 1.77 (0.25, 5.58)	0.4320
		Retract 3.06 (0.28, 5.62)	**Fast Strike vs. Ret**
			0.2700
Slow No bag	4 subjects	Strike 3.44 (0.72, 6.54)	**Fast vs. Slow**
		Retract 2.07 (0.31, 4.50)	2.7745e-015
			**Slow Strike vs. Ret**
			7.9882e-005
Fast No bag	4 subjects	Strike 4.20 (1.04, 5.38)	**Fast Strike vs. Ret**
		Retract 3.24 (1.59, 5.35)	0.1857

The Wilcoxon ranksum test for equal medians was performed to compare between strikes and retractions within each speed level. The comparison between speed levels was also performed.

**Figure 4:**
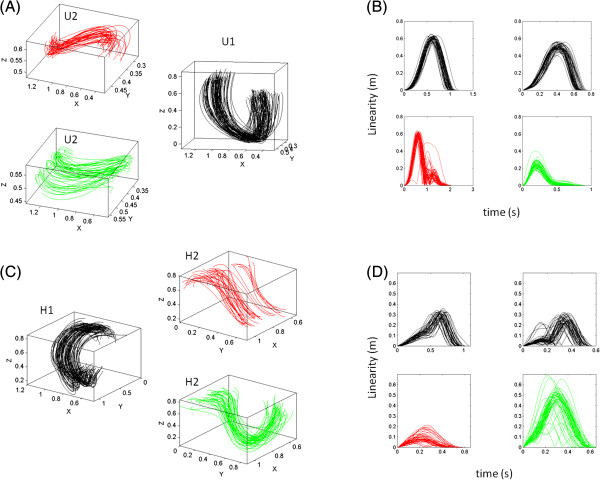
**Sample hand kinematics of the complex sequences in which the Jab was embedded: Conservation vs. non-conservation of trajectories according to changes in body dynamics (speeds and loads).** (**A**) The intended Uppercut motions U1 performed at different speeds maintain the curvature of the trajectories despite the changes in body dynamics. (**B**) In marked contrast the speed changes separate the curvatures of the spontaneous retracting segments, measured through a simple linearity metric (see Methods). (**C**)-(**D**) Similar behavior was registered for the Hook under speed and loads condition. Notice that the addition of loads makes the linearity more variable.

During the intended strikes the effects of instructed speed were less marked in the curvature of the trajectories, which maintained their linearity for the Jab and Cross. Even for the more complex Hook and Uppercut trajectories the effects of speed and loads during intended segments were negligible relative to those on the spontaneous retractions. The latter segments significantly changed their linearity with speeds and loads (Kruskal Wallis comparison of linearity fast vs. slow in retractions, *p < 10*^*-5*^). Examples of the trajectories from the intended strikes in the Uppercut (U1) are shown in Figure 4A along with examples of the spontaneous retraction segments (U2). Notice the differences in the orientation of the curve as well, and on the bending of it. The linearity index is shown in Figure 4B for the U1 and U2 segments.

The trajectories of the Hook forward (H1) and retracting hook (H2) are also shown for slow vs. fast in Figure 4C when the subject was wearing loads on the forearm. Notice in the corresponding linearity profiles in Figure 4 D that despite the loads the intended profiles for slow and fast do not significantly differ (*p > 0.3*) but they do markedly change with speed and loads in the retractions (*p < 10*^*-4*^).

Possible effects of fatigue were assessed in different contexts by comparing the maximum velocity in the first 10 and the last 10 trials within a block of 100 trials. The duration of the motion was also assessed for same-speed blocks. No significant differences were detected in the speed maxima of the Jabs’ strikes (Friedman’s non-parametric ANOVA *df*_1,8_*χ*^2^ 0.76, *p > 0.38*). No significant differences were detected in the speed maxima of the Jabs’ retractions either (*df*_1,8_*χ*^2^ 0.04, *p > 0.85*). The movement duration was not significantly affected in the Jabs’ strikes independent of the instructed speed, slow *χ*^2^ 0.04, *p > 0.85*, fast *χ*^2^ 2.29, *p > 0.13.* Yet the duration of the fast retractions was significantly different between the first 10 and the last 10 trials even though the instructed speed was the same within that block *(χ*^2^ 17.66, *p < 10*^*-5*^*),* but not significant in the slow case, *χ*^2^ 2.62, *p > 0.11.* Thus effects of fatigue may be detectable in the deceleration phase of the retracting fast motions and contribute to the noise component of the retracting action.

## Velocity-dependent stochastic rule

The training sessions are studied as a stochastic process. The distribution of maximum velocities is fit by a two-parameter log-normal distribution [31]. We set to uncover a first-order stochastic rule that connects the velocity spread of the current trial to the maximum velocity and acceleration of the previous trial. This relation is hypothesized to remain stable while learning occurs. The random parameters of interest assessed over time were the maximum speed and maximum acceleration. We also asked if anticipatory/learning strategies thus determined differed between intended and spontaneous motions.

We obtained a rule to parameterize the fluctuations of the speed and acceleration maxima within a stochastic framework. We followed the stochastic trajectory of the velocity-dependent parameters over 100 points (100 trials) of these micro-movements as the hand motions unfolded during training sessions where speed was randomly instructed, from trial to trial.

In this context we wanted to know the extent to which the system would have correctly updated the slow vs. fast velocity in an impending trial, based on the kinesthetic sensing of its change (the acceleration) from a previous trial –despite the random instruction. To this end I examined the noise of the scatter of points according to a stochastic rule. It is important to notice that this rule is only used to examine the evolution of the noise. It is not stated as the updating rule for these motions but rather used as a tool to gain insights on the noise as a function of learning and training context. Velocity and acceleration are co-dependent parameters. Thus their noise is expected to co-vary. Any split in this process which is systematically modulated by training context or instructed speed could be informative of anticipatory strategies. In this sense the spontaneous retractions were more interesting, as they are not explicitly instructed, occur rather fast and automatically, and coexist with other intended strikes. Thus any potential changes in the noise can be of great interest, particularly given the recent findings that retractions in these motions are the most informative segments [31] because the changes in dynamics alter the geometry of their trajectories (see also Figure 4).

Congruent with the behaviors of the motion trajectories, during the training sessions the subjects performing these routines split the trajectories of the hand during the retracting motions as a function of instructed speed. Across random repeats different speeds were identified with different curvatures along the complex sequences. Here once again this split was quantified with speeds (Figure 4A-B) and loads (Figure 4C-D) for the retractions of the Uppercut and Hook respectively. However in the strike segments of the same routines this was not the case. Were the underlying structures of the noise different in forward and retracting segments of the Jab? Recall that the Jab was performed embedded in the sequence and also in isolation.

The trials were taken in the order in which they were acquired and plotted according to the rule below. Fitting errors were quantified (Table 2) in both intended and incidental segments.

**Table 2:** Regression fit for expert and novices

	**Linear Polynomial model: f(x)** = **p1** * **x** + **p2**	
	**Intended**	**Incidental**
Expert Slow	Coefficients (with 95% confidence bounds):	Coefficients (with 95% confidence bounds):
	p1 = 0.6918 (0.6467, 0.7369)	p1 = 0.8846 (0.8516, 0.9177)
	p2 = 1.844 (1.679, 2.01)	p2 = 0.9094 (0.7559, 1.063)
	Goodness of fit:	Goodness of fit:
	SSE: 0.1239 R-square: 0.9303	SSE: 0.03596 R-square: 0.974
	Adjusted R-square: 0.9294 RMSE: 0.04206	Adjusted R-square: 0.9736 RMSE: 0.02175
Expert Fast	p1 = 0.6879 (0.6603, 0.7156)	p1 = 0.9852 (0.9792, 0.9912)
	p2 = 1.791 (1.694, 1.889)	p2 = 0.1223 (0.09412, 0.1504)
	SSE: 0.1045 R-square: 0.9723	SSE: 0.0009422 R-square: 0.9993
	Adjusted R-square: 0.9719 RMSE:	Adjusted R-square: 0.9993 RMSE:
	0.03863	0.003521
Novice 1 Slow (lacrosse expert in Figure 1)	p1 = 1.01 (0.9876, 1.038)	p1 = 1.23 (0.9936, 1.266)
	p2 = 0.95 (0.8598, 1.042)	p2 = 1.04 (0.9049, 1.176)
	SSE: 2.861 R-square: 0.9851	SSE: 1.704 R-square: 0.9857
	Adjusted R-square: 0.9849 RMSE: 0.1717	Adjusted R-square: 0.9854 RMSE: 0.1884
Novice 1 Fast	p1 = 1.03 (0.9936, 1.066)	p1 = 0.93 (0.8982, 0.9597)
	p2 = 0.87 (0.7878, 0.9687)	p2 = 1.03 (0.9539, 1.117)
	SSE: 1.704 R-square: 0.9857	SSE: 1.407 R-square: 0.9764
	Adjusted R-square: 0.9854 RMSE: 0.1884	Adjusted R-square: 0.9762 RMSE: 0. 1272
Novice 2 Slow (swimmer)	p1 = 1.31 (1.144, 1.478)	p1 = 1.37 (1.225, 1.515)
	p2 = 1.34 (1.268, 1.428)	p2 = 1.41 (1.339, 1.484)
	SSE: 0.9911 R-square: 0.9062	SSE: 0.5253 R-square: 0.933
	Adjusted R-square: 0.9027 RMSE: 0.1916	Adjusted R-square: 0.9305 RMSE: 0.1395
Novice 2 Fast	p1 = 1.43 (1.289, 1.58)	p1 = 0.82 (0.7393, 0.903)
	p2 = 1.23 (1.167, 1.296)	p2 = 1.56 (1.49, 1.635)
	SSE: 3.114 R-square: 0.8725	SSE: 0.8399 R-square: 0.9378
	Adjusted R-square: 0.8702 RMSE: 0.2337	Adjusted R-square: 0.9356 RMSE: 0.1732
Novice 3 Slow	p1 = 0.52 (0.4789, 0.5778)	p1 = 1.19 (0.5917, 1.797)
	p2 = 1.95 (1.851, 2.057)	p2 = 3.21 (0.7475, 5.677)
	SSE: 53.35 R-square: 0.7431	SSE: 4.943 R-square: 0.5245
	Adjusted R-square: 0.7414 RMSE: 0.5886	Adjusted R-square: 0.4948 RMSE: 0.5558
Novice 3 Fast	p1 = 0.47 (0.3578, 0.499)	p1 = 0.16 (0.1126, 0.2258)
	p2 = 1.78 (1.61, 1.85)	p2 = 5.63 (5.326, 5.941)
	SSE: 5.432 R-square: 0.8184	SSE: 0.3084 R-square: 0.7004
	Adjusted R-square: 0.8158 RMSE: 0.2806	Adjusted R-square: 0.6827 RMSE: 0.1347
Novice 4 Slow	p1 = 0.595 (0.5286, 0.6217)	p1 = 0.967 (0.9576, 0.9763)
	p2 = 1.151 (1.022, 2.281)	p2 = 0.217 (0.1741, 0.2594)
	SSE: 0.2796 R-square: 0.928	SSE: 0.001278 R-square: 0.89
	Adjusted R-square: 0.9265 RMSE: .07633	Adjusted R-square: 0.85 RMSE: 0.005161
Novice 4 Fast	p1 = 0.650 (0.6297, 0.7311)	p1 = 0.725 (0.7147, 0.7361)
	p2 = 1.901 (1.671, 2.131)	p2 = 1.767 (1.727, 1.806)
	SSE: 0.04217 R-square: 0.9382	SSE: 0.3144 R-square: 0.9546
	Adjusted R-square: 0.9369 RMSE: 0.02964	Adjusted R-square: 0.9346 RMSE: 0.05664
Novice 5 Slow	p1 = 0.441 (0.3199, 0.5625)	p1 = 0.501 (0.417, 0.5265)
	p2 = 4.616 (4.519, 4.714)	p2 = 4.653 (4.614, 4.693)
	SSE: 0.04739 R-square: 0.776	SSE: 0.06301 R-square: 0.8946
	Adjusted R-square: 0.7628 RMSE: 0.0528	Adjusted R-square: 0.8916 RMSE: 0.04184
Novice 5 Fast	p1 = 0.450 (0.4087, 0.4926)	p1 = 0.400 (0.27, 0.5318)
	p2 = 4.654 (4.6280, 4.6800)	p2 = 4.425 (4.317, 4.533)
	SSE: 0.007848 R-square: 0.9679	SSE: 0.03337 R-square: 0.7106
	Adjusted R-square: 0.966 RMSE: 0.02149	Adjusted R-square: 0.6936 RMSE: 0.0443

Across subjects speed ranged between 0.97 and 7.91 m/s in the intended segments and between 0.60 and 4.96 m/s in the spontaneous segments incidental to the strikes.

For each Jab sub-movement we set the velocity in the next trial proportional to the acceleration and the velocity of the current trial by approximating

$$\begin{array}{c} \left(A_{\max}^{t}+\upsilon \cdot V_{\max}^{t}\right)=\left(V_{\max}^{t+1}-V_{\max}^{t}+\upsilon \cdot V_{\max}^{t}\right)\\ \;=\left(V_{\max}^{t+1}+\nu \cdot V_{\max}^{t}\right) \end{array}$$

The constant of proportionality *υ* = 10, *v* = 1 – *υ* was obtained from the entire data set according to the ranges of velocity/acceleration values spanned across all participants. This was done to avoid only covering a subset of values spanned by any one given participant.

Here we take the minute fluctuation in the value of the maximum speed from trial to trial as a *micro-movement*. We then examine the variability of this parameter over the time course of a training session across over 100 trials in contrast to taking the actual motion trajectory of one trial.

Notice that somatosensation and the kinesthetic percept of movement –which are related to our read out in these micro-movements- is particular to each individual. Each person spans a different velocity-dependent stochastic signature, so this rule has different parameters for each person. The normalization υ = 10 pertains only to the range of values of the parameters of interest (velocity and acceleration maxima) so that we speak of a scale common to all subjects in the database. The constant will be different in other cases (e.g. infants or elderly with a compromised system, etc. given the range of values of a given cohort).

The previous work revealed an exponential relation between the shape and scale parameters of the continuous Gamma probability distribution family. The frequency distribution of the log-transformed of the velocity-dependent data turned from skewed to normal, was well fit by the two-parameter log-normal distribution and fell in the symmetric ranges of the Gamma plane. A power fit of the scatter was obtained in our previous work to characterize the (log-shape, log-scale) scatter of the Gamma plane. Motivated by those results, here we use a linear model *f*(*x*) = *mx* + *b* + *ε* to characterize log-relations of the noise present in velocity-dependent measurements from trial to trial. The *m* is the slope of the line, *b* is the intercept and *ε* refers to the fit-error. In the case of the Jab, we consider the training context as the *task* and replacing the above approximation on the equation of the line gives,

(2) $$\left(V_{\max}^{t+1}\left(\mathrm{task}\right)+\nu \cdot V_{\max}^{t}\left(\mathrm{task}\right)\right)=m\left(A_{\max}^{t}\left(\mathrm{task}\right)\right)+b\left(\mathrm{task}\right)+\varepsilon \left(\mathrm{task}\right)$$

We then take the natural logarithm of the parameters of interest, and assume that the properties of the noise will change with the training context as well:

(2.1) $$\ln\left(V_{\max}^{t+1}\left(\mathrm{task}\right)+\nu \cdot V_{\max}^{t}\left(\mathrm{task}\right)\right)=m\ln\left(A_{\max}^{t}\left(\mathrm{task}\right)+b\left(\mathrm{task}\right)+\varepsilon \left(\mathrm{task}\right)\right)$$

The parameters of interest are the velocity and acceleration maximum in each trial which we have previously characterized using the continuous family of Gamma probability distributions with shape and scale parameters [32,37].

The log-normality of the velocity-dependent parameters [31] informs us of the empirical nature of the random process that we are studying: the behavior of the random parameter over time. The time scale that we are assessing is between 0.8 s and 1.7 s per trial. The experimental session under a given context has 100 trials (e.g. simulation of the routine from memory as if striking an imaginary opponent).

In the rule that we are testing for one session *t* and *t + 1* refer to the trial order number, ν is the same scaling factor for all subjects (as explained above), *m* and *b* are the slopes and intercepts of the best-fitting regression lines of the scatter, and ε is the residual error. The error-noise distributed normally across subjects for the intended strikes and its variance served to track learning in each subject under a given condition across all randomly called speed levels. Table 2 reports root mean squared fitting errors for the 6 subjects that were involved in the various manipulations of sensory guidance.

## Exponentiation of (1.1) gives

$$\begin{array}{c} e^{\ln\left(V_{\max}^{t+1}\left(\mathrm{task}\right)+\nu \cdot V_{\max}^{t}\left(\mathrm{task}\right)\right)}=e^{m\ln\left(A_{\max}^{t}\left(\mathrm{task}\right)\right)+b\left(\mathrm{task}\right)+\varepsilon \left(\mathrm{task}\right)}\\ \;=e^{m\ln\left(A_{\max}^{t}\left(\mathrm{task}\right)\right)}e^{b\left(\mathrm{task}\right)+\varepsilon \left(\mathrm{task}\right)} \end{array}$$

Using the logarithmic and exponent rules *a*^*m* + *n*^ = *a*^*m*^ · *a*^*n*^ and *a* = *e*^ln *a*^,  *a*^*x*^ = (*e*^ln *a*^)^*x*^ = *e*^*x* ln *a*^ for each real *x* and if *a*^*x*^ is to preserve the logarithmic and exponent rules,

(2.2) $$\begin{array}{l} e^{m\ln\left(A_{\max}^{t}\left(\mathrm{task}\right)\right)}e^{b\left(\mathrm{task}\right)+\varepsilon \left(\mathrm{task}\right)}=e^{{\left(\ln\left(A_{\max}^{t}\left(\mathrm{task}\right)\right)\right)}^{m}}e^{b\left(\mathrm{task}\right)+\varepsilon \left(\mathrm{task}\right)}\\ V_{\max}^{t+1}\left(\mathrm{task}\right)+\nu \cdot V_{\max}^{t}\left(\mathrm{task}\right)={\left(A_{\max}^{t}\left(\mathrm{task}\right)\right)}^{m}e^{b\left(\mathrm{task}\right)+}{}^{\varepsilon \left(\mathrm{task}\right)} \end{array}$$

For example the fitting parameters for a novice were *m = 1.03, b = 0.87*, correlation coefficient *0.98* for the intended fast Jab (Table 2, lacrosse player novice at martial arts.) From the slope value which we can write as *m = 1 – δ*, we can approximate equation (2.2) to leading order as:

$$\begin{array}{c} V_{\max}^{t+1}\left(\mathrm{task}\right)=A_{\max}^{t}\left(\mathrm{task}\right)\left[1-\delta \ln A_{\max}^{t}\left(\mathrm{task}\right)+O\left(\delta^{2}\right)\right]\\ \;\cdot e^{b\left(\mathrm{task}\right)+}{}^{\varepsilon \left(\mathrm{task}\right)}-\nu \cdot V_{\max}^{t}\left(\mathrm{task}\right) \end{array}$$

with *δ = −0.03* implying a stochastic updating-rule that anticipates the maximum speed (event at trial n) in the upcoming trial n + 1 based on the combination of current maximum speed and maximum acceleration of the current trial with multiplicative error.

Notice here that we use this rule to characterize the movement across different contexts and expect changes in the slope; intercept and error (scatter) as a function of context, effort, fatigue etc. Yet we wanted to know if within one task context the scatter maintained this first order stochastic rule. Also notice that in light of a range of maximum speeds between 0.25 and 9 m/s the squared absolute value of *0.03*^*2*^*, (9.0 x 10*^*-4*^*)* affecting the slope of the scatter can be considered as negligible.

**Figure 5:**
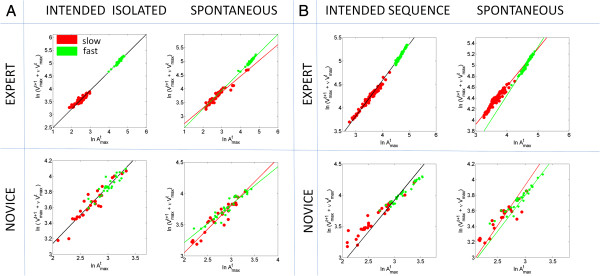
**Anticipatory performance of the expert vs. representative novice participant across different training sessions.** The scatter is comprised by the trials from fast and slow speed according to the first order rule used to parameterize the relation between the maximum velocity and acceleration from trial to trial. (**A**) Isolated Jab trials performed at different speeds for intended and spontaneous segments form self-aggregates. Top is from the expert and bottom from the representative novice 1 in Table 2. (**B**) Performance from a subsequent session where the participants executed the Jab embedded in the full fluid sequence. Notice the improvement in the novice upon training whereby the Jab embedded in the complex sequence begins to cluster correctly as a function of instructed speed. Notice also that spontaneous movements “channeled” out through a different slope the type of instructed speed.

In Figure 5 the expert’s speed-dependent data and the speed-dependent data from a representative novice are plotted according to this rule for all trials in one session. Notice that the expert’s motions can self-segregate fast from slow in both intended and incidental segments. The scatters from the intended segments can be well fit with a single slope for both speeds but this is not the case for the incidental motions. The latter require two different exponents for a good fit. This splitting feature in the noise from the spontaneous retractions as a function of speed level is observed in both the isolated Jab and the Jab embedded in the full sequence, albeit more variable in the sequence case.

The novices tended to generally behave similarly to the expert in the intended strikes. However, not surprisingly their motions were more variable and characterized by more errors in the prediction of impending speed type from previous trial maximal acceleration and speed combination (see Table 2). In marked contrast to the expected expert behavior in the intended motions in several novices more than one slope were necessary to fit the instructed speed-driven noise.

In the segments incidental to the strike all novices required two different slopes to fit well the scatters from the two speed levels. In other words, the spontaneous retractions funneled out the speed type through two different slopes and intercepts, a result that stood regardless of expertise level.

**Figure 6:**
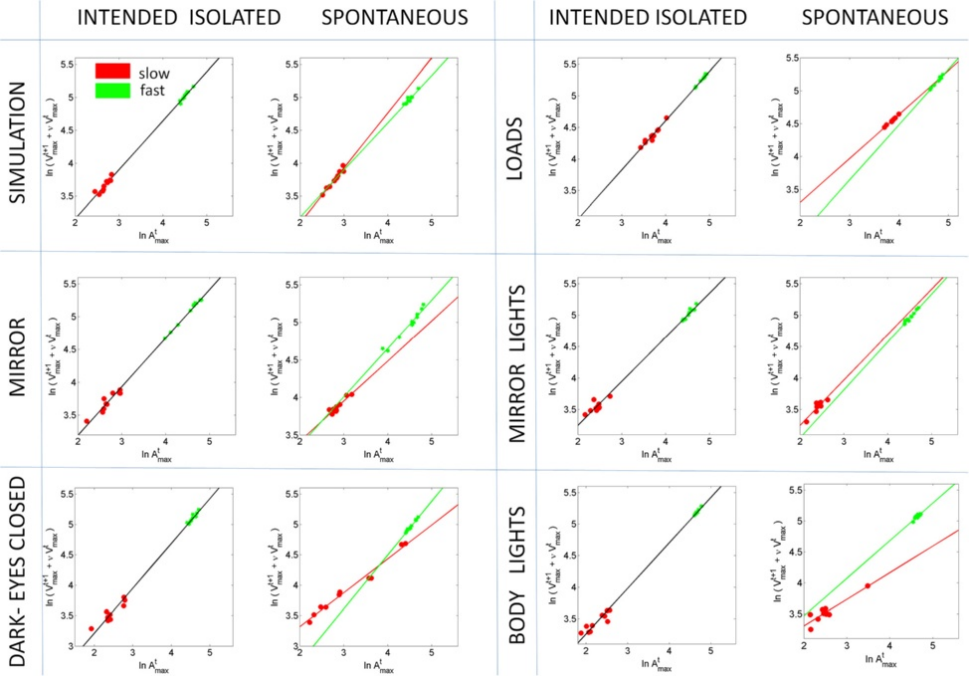
Systematic effects of speed level and training context on the noise properties of the spontaneous retractions in the expert system.

In 6 participants we examined the two sub-movements of the Jab under different contexts. As in the case of the simulation condition, under other training contexts we found that the spontaneous retraction always separated the noise according to instructed speed type. In the expert motions shown in Figure 6 we also found that the slopes and intercepts across training contexts remained similar when the Jab was intended to strike a target (imaginary) opponent. However in the spontaneous retractions the noise split not only as a function of instructed speed but also the slope and intercept systematically changed as a function of training context. These spontaneous motions funneled out the randomly instructed speed type and the differences in training context (dark, mirror, etc.) as well. In novices the slope of the intended scatter was not as stable as that of the expert’s case. The tilt of the intended slope changed with the context. Yet the spontaneous retractions did split the noise differently as a function of speed and context, regardless of expertise level.

Expertise level in general sports also played a role (not surprisingly). During the intended strike segments the novices with expertise in other sports tended to behave similarly to the expert but those who were not athletes tended to require two lines for a better fit. Figure 5 (novice) depicts higher variability and dispersion of the scatter. Yet, notice that a single slope fits well the scatters from the intended strikes; whereas two distinct slopes fit the spontaneous retractions (see Table 2 for data from all subjects in this group).

## Distributional analyses of velocity-dependent parameters

**Figure 7:**
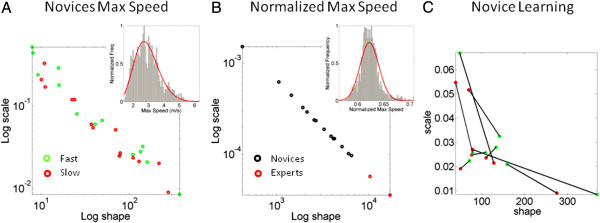
**The velocity-dependent parameters reveal learning according to the subject’s somatosensory stochastic signatures in each training context.** (**A**) The empirical frequency distribution of maximum speed across subjects (inset) and the MLE of shape and scale Gamma parameters for each subject, for the fast and slow instructed speed condition. (**B**) The normalized maximum speed parameter (invariant to possible allometric effects due to individual differences in limb sizes) aligns participants across the line of unity with experts at the far right symmetric range of the Gamma (more predictive power in their performance). Inset shows the empirical frequency distribution across subjects for this normalized parameter. (**C**) The individual learning progression for novices as they performed slow and fast versions of the jab. Notice that the stochastic signatures of their speed maxima shifts towards the right for the fast condition (more predictive) in some cases, whereas in other cases it is instead the slow condition which has this effect. Notice also that given the same number of repetitions, the rate of change is very small for some subjects and very large for others. This plot captures the individual’s learning progression and unveils which training context is most adequate to make the subject’s motions more predictable.

As in our previous work, we did not assume anything about the underlying probability distribution governing these random processes. Instead, we actually estimated the statistics from the empirical frequency distributions of the parameters of interest. As previously, here we found that these empirical distributions were not symmetric. The parameters of the continuous Gamma family of probability distributions served to characterize with high confidence the shape and scale of the empirical frequency distributions of the maximum velocity. Figure 7A shows an example of a representative empirical (normalized) frequency histogram with the fit from the Gamma parameters (inset). The red and green dots on the Gamma plane represent the labeling of (shape, scale) parameters estimated for each novice participant. For simplicity we do not show the confidence intervals. Notice that subjects in the upper left corner of the Gamma plane (5 novices) are the group where within a block they received randomly the instructed speed level, whereas subjects towards the right participated in the block design. Not surprisingly, the latter have patterns towards the (more predictive) symmetric range of the Gamma. This speed parameter highlights individual stochastic differences in performance for different speed levels. In Figure 7B one can see the ensemble behavior independent of limb size effects (using the normalized maximum speed metric.) Notice there that the two experts stand apart from the novices and that the frequency histogram is not as skewed as in A. This normalization is important when comparing subjects of different ages, sizes and gender. Figure 7C shows the individual progression based on the speed maxima for different speed blocks in the second group of novices. Notice there that for some participants the shift in stochastic signature was very large from the slow to the fast block, whereas for others it was a modest shift. Likewise in some cases the fast condition made them more predictive (towards the right) whereas in others it made their patterns more random (towards the left). This graph shows the advantage of this method to personalize training regimes while assessing predictability in real time.

### Analyses of speed variability under different training conditions: hitting a physical target vs. hitting an imaginary target

**Figure 8:**
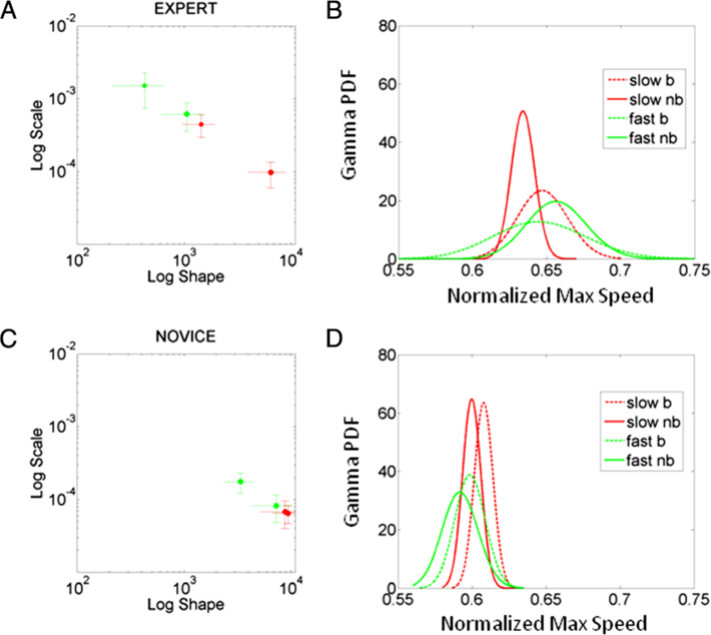
**Statistics of the normalized maximum speed labeling subjects on the Gamma plots for representative novice and expert.** (**A**) Expert (a,b) MLE for each speed condition and training context (bag vs. no-bag) with 95% confidence intervals. (**B**) The corresponding Gamma probability density function (PDF) curves reveal in the expert a broad bandwidth of parameter values across training contexts. It also shows an unambiguous distinction between bag and no-bag conditions for each speed level. Speed levels are not confused by the expert’s kinesthetic data. (**C**-**D**) The novice however shows a narrow bandwidth of parameter values with no clear distinction between slow motions that are against the bag or towards a simulated opponent. The novice’s kinesthetic data does distinguish between the fast-bag condition and the other training contexts. Notice the degree of dispersion of the probability distribution measured through the Fano Factor (noise to signal ratio) $F\;=\;\frac{\sigma_{w}^{2}}{\mu_{w}}$ the ratio of variance to mean taken within the time window *w* (the time in ms to reach the peak velocity, on the order of 200 ms in this case) is indistinguishable in the novice for the slow case (6.47 × 10^-5^ slow-bag vs. 6.30 × 10^-5^ slow-no-bag) and for the fast case (1.76 × 10^-4^ fast-bag vs. 2.47 × 10^-4^ fast-no-bag). The novice can however differentiate between fast and slow (Wilcoxon ranksum test of equal medians p < 10^-3^). Compare to the expert with Fano factors that distinguished speed within each training context (slow-bag 4.4 × 10^-4^ vs. fast-bag 0.0015; slow-no-bag 9.7 × 10^-5^ vs. fast-no-bag 4.08 × 10^-4^).

In a small subset of participants (2 experts and 2 novices) we assessed the ability of their somatosensory patterns to distinguish speed levels and training conditions. To this end we estimated the Gamma parameters and using their empirical range of values for the normalized maximum velocity, we obtained estimates of the probability density function curve corresponding to each condition. Then we obtained the statistics of the random parameter according to the Gamma pdf (*a* is the shape and *b* is the scale, the Expected value and Variance are E[X] = *a.b* and Var[X] = *a.b*^*2*^). We then computed the Fano Factor (noise to signal ratio) $F\;=\;\frac{\sigma_{w}^{2}}{\mu_{w}}$, the ratio of variance to mean taken within the time window *w* (the time in ms to reach the peak velocity, on the order of 200 ms in this case) for each case. The Figure 8A shows the expert performance. One can see that the expert distinguishes between speed levels very well, but also it distinguishes within each speed level whether the strike was intended to hit a physical opponent in the form of an actual punching bag, or an imaginary one (simulated condition) with no punching bag. Notice as well that the expert spans a broad bandwidth of values and has low noise to signal ratio in all conditions (reported in the caption). By marked contrast the novice’s somatosensation cannot distinguish whether the slow speed is from the bag or no-bag case and the fast case is also confusing, albeit less confusing than the slow. His speed fluctuations can however generally separate between fast and slow trials between blocks. Yet the bandwidth of values that he samples from is still very narrow and has higher noise to signal ratios than the expert. In this sense expertise has two critical ingredients: (1) low noise to signal ratio that can blindly separate within speed levels the different training regimes; and (2) a broad bandwidth of values for the given parameter. Although 4 subjects is a small sample size, we just wanted to illustrate the statistical technique, which is only dependent upon the individual’s somatosensation readout from the fluctuations of the micro-movements as a stochastic process over time. This technique can be also used to assess the performance of a team.

## Conclusions

This work studied the statistics of velocity-dependent parameters from the hand movement trajectories of novices and experts at martial arts routines as their motions unfolded. Several training contexts were used to investigate the statistical signatures of intended strikes and spontaneous retractions of the Jab. The empirical frequency histograms of the maximum speed were obtained and the continuous Gamma family of probability distributions was used to estimate the statistical properties of the fluctuations of the maximum speed. We show that these distributions change on demand with the training context. They should therefore not be assumed as homogeneous for all subjects and conditions when studying natural movements. Furthermore it was corroborated that the empirically estimated parameters of the distribution differed between intended and spontaneous movement classes. Not surprisingly the intended motions differed between experts and novices –as they are specifically trained to do. Yet unexpectedly, learning revealed larger differences for different speeds and training contexts in the spontaneous retractions. These supportive motions automatically occur without instructions and largely below awareness as the system is deliberately tending to the instructed strikes. The findings show a statistical distinction between intended and automatic motions which may be of relevance for sports science and also for clinical research. The results on different training contexts and instructions on the use of visual feedback also suggest that the distinction between intended and unintended motions can flexibly change and affect the movement statistics. This result has implications for sports training.

We used a speed-acceleration dependent first order stochastic map to describe the noise of the performance across a session. This stochastic relation, which combined the previous trial velocity and acceleration, predicted maximum velocity of the current trial. The decay parameters of the best fitting relation, when plotted across subjects and as a function of expertise, was well characterized by a power law. The power law exponent was generally similar for fast and slow intended strikes, but always different for incidental strikes.

As in our previous work we confirmed that intended trajectories are more robust to changes in dynamics -such as combinations of speeds and loads- than trajectories in the spontaneous retractions. The latter changed dramatically their geometric properties with changes in dynamics. These effects were also reflected in the noise of velocity-dependent parameters which split during retractions according to the training context. In this sense such supportive movements are surprisingly more informative than their intended counterparts when they are not instructed. Under instruction the retractions cease to be spontaneous and tend to lose their distinction from the strikes. An open question is what training regime (instructed or spontaneous) suits a given athlete best. The proposed metrics can be used to measure the statistical properties of such distinctions.

The present findings alert us of the potential importance of re-afferent kinesthetic input coming to the central nervous system from peripheral nerves and autonomic centers. The new framework and methodology that we offer here could be used to assess sensory-motor processes below awareness that may nonetheless contribute to the emergence of somatosensation as a verifiable percept in a new context to facilitate anticipatory behavior and learning. Such a percept would be useful for automatic brain-body interactions operating at faster timescales than deliberate processes, once a level of expertise has been attained. In the context of Bayesian inference models such a reliable somatosensoty percept would constitute an acquired kinesthetic “prior” that would shift on demand at different time scales according to expertise level.

This work also assessed the trajectories of the subject’s stochastic signatures over time and provided new tools to assess performance levels in terms of velocity dependent predictability. In particular it was shown how to assess learning in terms of the statistics of the micro-movements by empirically estimating a probability distribution rather than assuming one. Indeed, the probability distributions that were empirically derived markedly changed from subject to subject, inviting new methods of statistical analyses for sports and movement science. A set of criteria for expertise was unveiled as well which included (1) lower noise to signal ratios in the dispersion of the distribution; (2) distinct “priors” for each speed, context and speed-context combination; (3) broad bandwidth of parameter values. This combination of diversified somatosensory re-afferent input and low noise to signal ratio makes the expert’s kinesthetic percept more reliable than that of the novice. It also points at ingredients to describe automaticity in statistical terms according to a veritable kinesthetic percept with high predictive value that can unambiguously distinguish not only the speed level of the motion, but also the speed level that a given context may call for. Such flexibility in adapting to different training regimes was what distinguished the expert performance above and beyond the obviously more variable performance of the novice. Lastly the novice’s spontaneous retractions were informative too despite level of expertise in other sports, but their intended strikes were closer to those of the expert only for those who practiced other sports.

We suggest that intended motions may be a predictor of overall expertise level whereas incidental motions may serve to track the progression of learning to kinesthetically reliably discriminate the patterns of fluctuations associated with changes in movement speed. This work reveals an important role for the supportive task-incidental motions. These movements that “glue” together our goal-directed behaviors could be revealing of subtle learning strategies supporting volitional control. We should turn our attention to such motions as they remain largely below awareness but make up a large portion of our natural behaviors.

## Competing interest

The author declares that she has no competing interest.

## Author’s information

Elizabeth B Torres: https://sites.google.com/site/sensorymotorintegrationlab/projects/from-voluntary-to-automated-control.
